# Beyond Hypertrophy: A Case of Mid-ventricular Obstructive Hypertrophic Cardiomyopathy With Apical Sequestration

**DOI:** 10.7759/cureus.99515

**Published:** 2025-12-18

**Authors:** Oumaima Taoussi, Soukaina Scadi, Marwa Mokhtari, Ghali Benouna, Fatimazahra Merzouk

**Affiliations:** 1 Cardiology, Mohammed VI International University Hospital, Mohammed VI University of Health Sciences (UM6SS) and Mohammed VI Foundation of Health Sciences, Casablanca, MAR; 2 Cardiology, Cheikh Khalifa International University Hospital, Mohammed VI University of Health Sciences (UM6SS) and Mohammed VI Foundation of Health Sciences, Casablanca, MAR

**Keywords:** apical sequestration, apical thrombus, cardiac magnetic resonance, hypertrophic cardiomyopathy, ischemic injury, mid-ventricular obstruction, multimodality imaging

## Abstract

Apical sequestration is an often overlooked manifestation of mid-ventricular obstructive hypertrophic cardiomyopathy (MVO-HCM), in which dynamic mid-ventricular obstruction creates an anatomically and functionally isolated apical chamber with impaired distal perfusion. We describe a patient with MVO-HCM who developed apical sequestration complicated by flow stagnation, ischemic injury, and a small mural apical thrombus. Transthoracic echocardiography showed marked mid-ventricular hypertrophy with systolic narrowing. At the same time, cardiac magnetic resonance provided a more comprehensive characterization by demonstrating a mechanically trapped apical chamber, mid-ventricular intramural fibrosis, a small apical thrombus, and transmural late gadolinium enhancement consistent with ischemic injury. Coronary angiography revealed no obstructive disease, supporting a mechanism of obstruction-related flow limitation rather than epicardial coronary pathology. This case underscores apical sequestration as a high-risk morphological and physiological variant of MVO-HCM. It highlights the value of multimodality imaging, particularly cardiac magnetic resonance, in identifying this uncommon presentation and guiding clinical management.

## Introduction

Mid-ventricular obstructive hypertrophic cardiomyopathy (MVO-HCM) is an uncommon morphological variant of hypertrophic cardiomyopathy in which pronounced mid-ventricular hypertrophy produces a dynamic systolic narrowing of the mid-cavity [1]. Although overall infrequent, this subtype is clinically important because it may present with exertional dyspnea, chest discomfort, palpitations, arrhythmias, or embolic events, features that can initially appear nonspecific and delay recognition. The mid-cavitary obstruction can generate a substantial pressure gradient between the mid-ventricular and apical segments, progressively isolating the left ventricular apex both functionally and hemodynamically [2]. As the distal cavity becomes confined, forward flow diminishes, and the apex behaves like a partially separated chamber exposed to abnormal loading and perfusion conditions.

Apical sequestration carries important clinical implications. Beyond reduced washout alone, a combination of disturbed flow patterns, elevated intracavitary pressures, altered diastolic filling, and compromised subendocardial perfusion may converge to create an ischemic-prone environment within the trapped apex. These pathophysiological changes also facilitate mural thrombus formation, even when the epicardial coronary arteries are unobstructed [3,4]. Despite these clinically significant risks, apical sequestration remains under-recognized in routine practice, in part because the distorted apical geometry may be inadequately visualized on transthoracic echocardiography, leading to missed early ischemic changes or unrecognized thrombus formation [5].

We report a case of MVO-HCM with apical sequestration in which cardiac magnetic resonance (CMR) enabled detailed characterization of the trapped apex, demonstrating early ischemic changes and identifying a small mural apical thrombus. This case illustrates the diagnostic value of multimodality imaging and underscores the importance of heightened clinical vigilance toward this distinctive anatomical pattern and its potential complications [6].

## Case presentation

History

A 67-year-old woman was admitted for evaluation of a transient ischemic attack (TIA) characterized by transient right-sided weakness and speech disturbance that resolved spontaneously within minutes. Neurological assessment, including CT and MRI of the brain, showed no hemorrhage or territorial infarction. She had no prior cerebrovascular disease, atrial fibrillation, or significant carotid stenosis.

During the preceding months, she had experienced exertional dyspnea and intermittent palpitations, gradually increasing in frequency but never previously investigated. She denied syncope, orthopnea, or chest pain. Her cardiovascular history was otherwise unremarkable.

The admission electrocardiogram revealed diffuse repolarization abnormalities, with deep T-wave inversions in the anterolateral leads that appeared disproportionate to her known medical history, raising suspicion of underlying structural heart disease.

Clinical examination

Physical examination revealed a grade 2/6 systolic murmur best heard along the left sternal border, suggestive of dynamic intraventricular obstruction. Blood pressure and heart rate were stable, oxygen saturation was normal, and no signs of pulmonary or peripheral congestion were present. Neurological examination was normal at the time of assessment.

Investigations 

**Figure 1:**
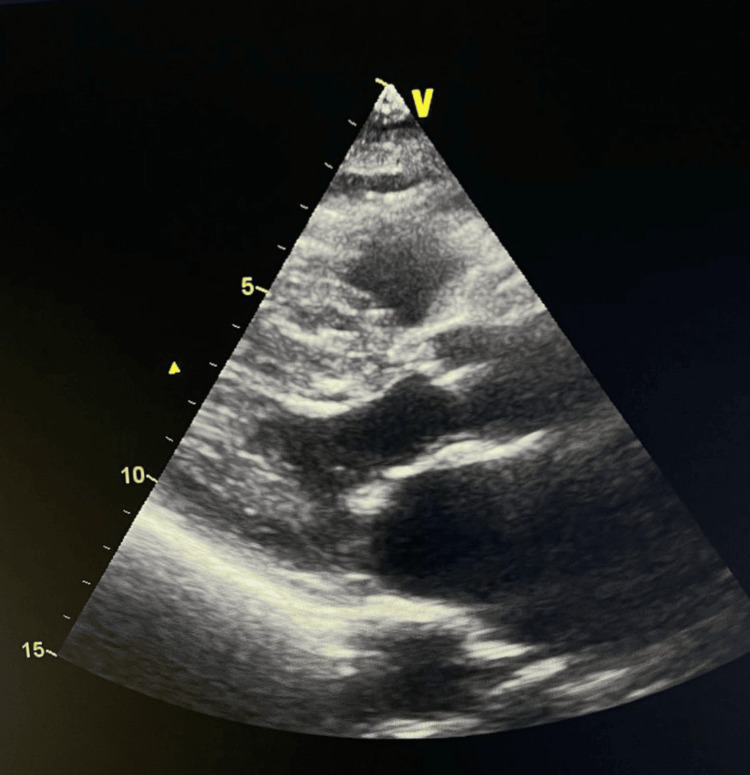
Parasternal long-axis view demonstrating ventricular hypertrophy. Parasternal long-axis (PLAX) transthoracic echocardiographic image showing marked left ventricular hypertrophy involving the mid-ventricular segments. Echogenicity is suboptimal, limiting precise visualization of the apical region, but the degree of hypertrophy is clearly appreciable. This pattern is suggestive of obstructive hypertrophic cardiomyopathy phenotype.

Transthoracic echocardiography demonstrated marked left ventricular hypertrophy involving the mid-ventricular segments. Image quality was limited due to suboptimal acoustic windows, making the apical region difficult to delineate and preventing reliable assessment of apical wall motion (Figure 1). The apex appeared hypokinetic but incompletely visualized.

**Figure 2:**
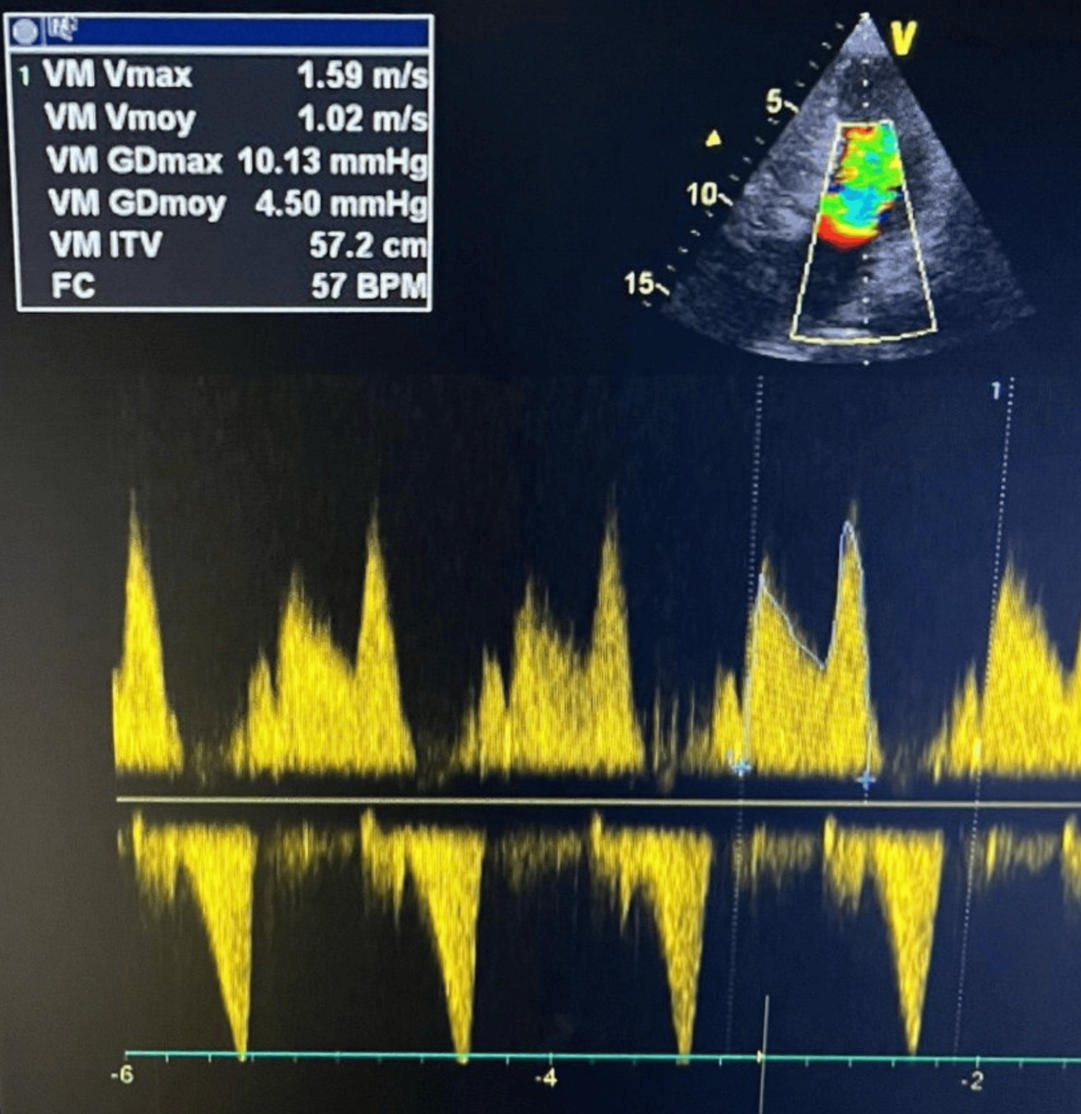
Continuous-wave Doppler revealing a systolic mid-cavity “saber-shaped” signal. Continuous-wave Doppler tracing recorded from the apical window showing a characteristic systolic mid-ventricular “saber-shaped” flow signal, reflecting a high-velocity gradient within the mid-cavity. This pattern is typical of mid-ventricular obstruction in hypertrophic cardiomyopathy.

Continuous-wave Doppler revealed a high-velocity systolic “saber-shaped” signal generated within the mid-cavity, reflecting a dynamic intraventricular pressure gradient characteristic of mid-ventricular obstruction (Figure 2). This Doppler contour, produced by systolic cavity narrowing and accelerated flow, is a well-recognized marker of mid-cavitary gradients in MVO-HCM. The gradient increased further during the Valsalva maneuver, confirming its dynamic nature and reinforcing the diagnosis of mid-ventricular obstruction.

**Figure 3:**
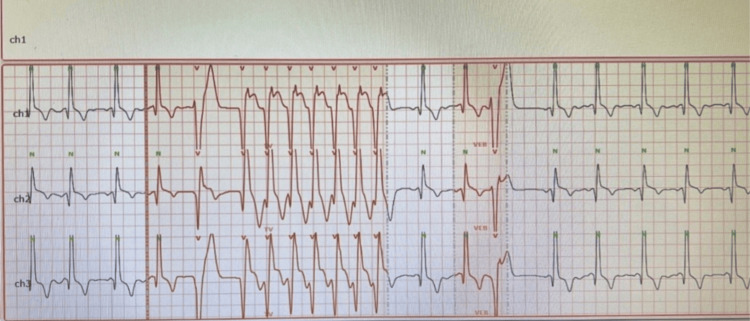
Holter monitor recording demonstrating non-sustained ventricular tachycardia. Twenty-four-hour Holter monitor strip showing an episode of NSVT consisting of several beats of a rapid monomorphic ventricular rhythm before spontaneous termination. NSVT: non-sustained ventricular tachycardia; VEB: ventricular ectopic beats (premature ventricular complexes).

A 24-hour Holter ECG documented an episode of non-sustained ventricular tachycardia (NSVT) without atrial fibrillation, supporting arrhythmic vulnerability within the context of suspected hypertrophic cardiomyopathy (Figure 3).

Coronary angiography was performed because the echocardiogram demonstrated apical hypokinesia and CMR suggested possible ischemic injury. Angiography showed strictly normal epicardial coronary arteries, ruling out obstructive coronary disease.

**Figure 4:**
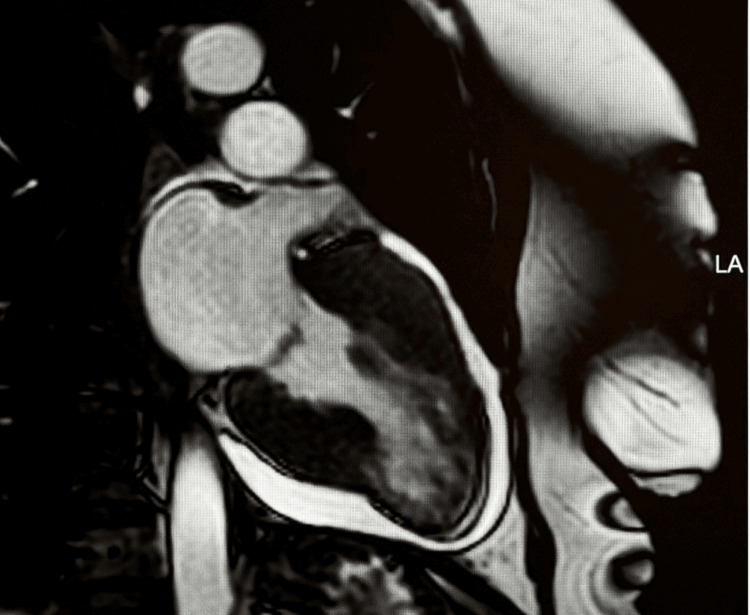
Two-chamber cine view demonstrating mid-ventricular hypertrophy. Cine steady-state free precession (SSFP) two-chamber view showing marked mid-ventricular hypertrophy with dynamic systolic narrowing of the mid-cavity, resulting in partial functional exclusion of the left ventricular apex. This configuration is characteristic of mid-ventricular obstructive hypertrophic cardiomyopathy.

To clarify the underlying pathology, the patient underwent cardiac magnetic resonance imaging. CMR confirmed severe mid-ventricular hypertrophy with dynamic systolic mid-cavity narrowing and functional exclusion of the left ventricular apex, consistent with mid-ventricular obstructive hypertrophic cardiomyopathy with apical sequestration (Figure 4).

Maximal wall thickness measured 28 mm in the mid-septal segment, 18 mm in the mid-lateral wall, and 15 mm in the mid-inferior wall, demonstrating asymmetric but pronounced involvement of the mid-ventricular level. The apex, although functionally isolated, did not display the features of a true apical aneurysm, as the apical myocardium remained thick, without focal thinning or dyskinesia, supporting the diagnosis of apical sequestration rather than aneurysm formation.

**Figure 5:**
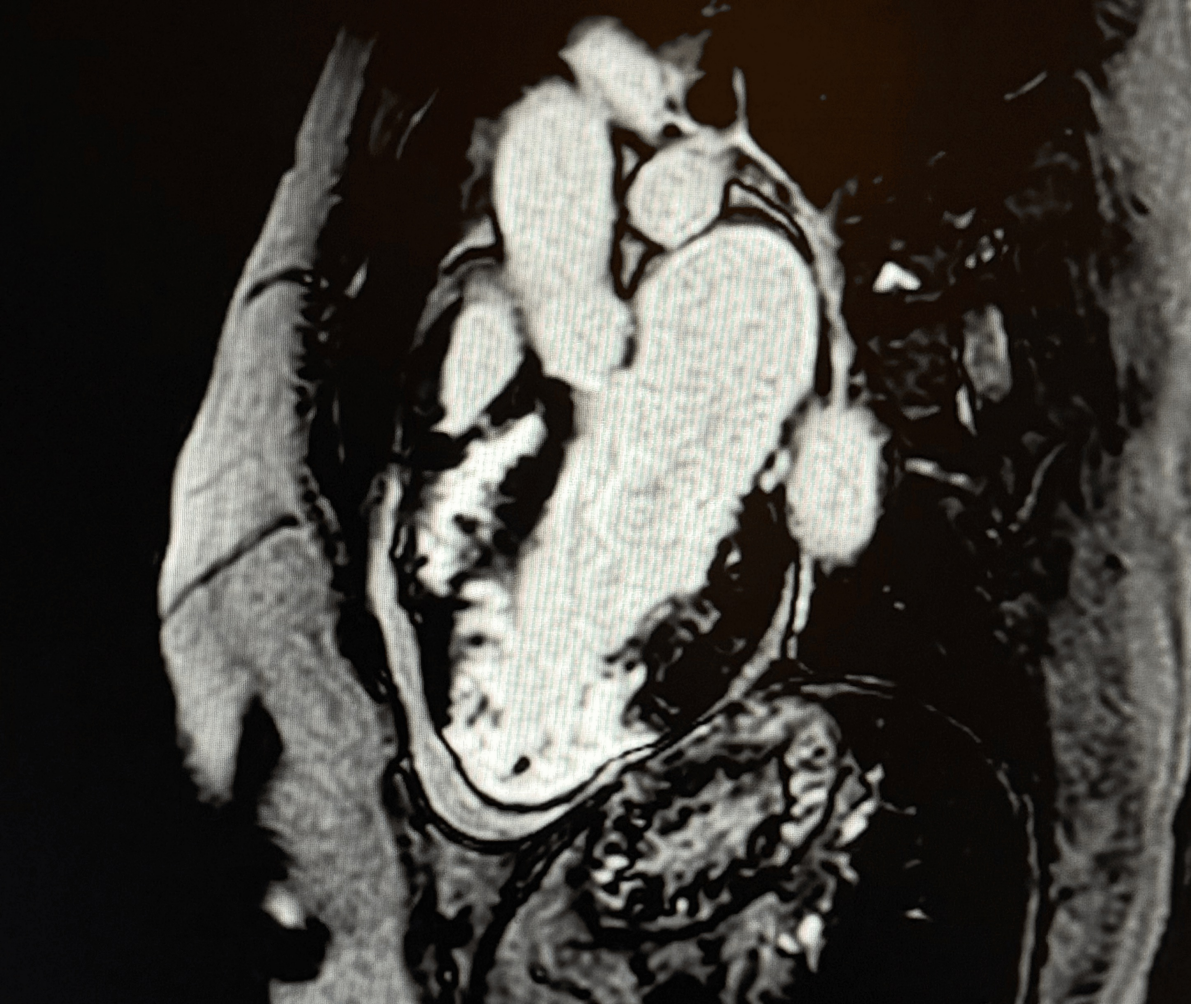
Three-chamber LGE image showing hypertrophic septum fibrosis, apical ischemia, and thrombus. Three-chamber late gadolinium enhancement (LGE) sequence demonstrating transmural enhancement of the apical wall, consistent with ischemic injury within the functionally isolated, sequestrated apex. Intramural enhancement is present in the mid-ventricular septal segments, reflecting fibrosis within the hypertrophied myocardium. The apex, although isolated, does not exhibit features of a true aneurysm, as the apical myocardium remains thick and without focal thinning. A small crescent-shaped apical mural thrombus is visualized as a dark signal void adjacent to the enhanced apical myocardium.

Tissue characterization showed extensive intramural fibrosis within the hypertrophied mid-ventricular segments, while the sequestrated apex exhibited transmural late gadolinium enhancement (LGE) consistent with ischemic injury resulting from chronic distal pressure overload and reduced washout rather than epicardial coronary disease. A small, crescent-shaped mural apical thrombus measuring approximately 5-7 mm was also identified as a dark signal void on LGE sequences (Figure 5).

Management

Management focused on preventing embolic events, reducing dynamic obstruction, and mitigating arrhythmic risk, and given the presence of an apical mural thrombus within a chronically low-flow sequestrated chamber, long-term anticoagulation was initiated with the intention of lifelong continuation unless contraindications arise, both to treat the existing thrombus and to prevent recurrent embolic complications, while beta-blocker therapy was introduced to attenuate the mid-ventricular gradient, improve diastolic filling, and reduce myocardial oxygen demand, and sudden cardiac death (SCD) risk stratification incorporated multiple high-risk markers including severe mid-ventricular hypertrophy, extensive fibrosis on CMR, functional apical isolation, and documented non-sustained ventricular tachycardia on Holter monitoring, all of which collectively supported the indication for an implantable cardioverter-defibrillator (ICD) for primary prevention, and ICD implantation was performed after shared decision-making.

Outcome and follow-up

Following ICD implantation the patient remained clinically stable with improvement in exertional dyspnea and no recurrence of palpitations, ICD interrogations revealed no ventricular arrhythmias, anticoagulation was continued without adverse effects, and because early CMR is not recommended shortly after device implantation a follow-up cardiac MRI was scheduled at approximately six months to reassess thrombus evolution, confirm stability of the sequestrated apex, and exclude complications such as aneurysm development, acknowledging that the pattern of late gadolinium enhancement is not expected to regress, and the patient continues guideline-directed therapy with regular clinical and device surveillance.

## Discussion

Mid-ventricular obstructive hypertrophic cardiomyopathy (MVO-HCM) is an uncommon but clinically significant variant of hypertrophic cardiomyopathy in which dynamic mid-cavity obstruction produces a high-pressure, functionally isolated apical chamber [7]. This configuration creates a unique hemodynamic environment that promotes ischemia, apical remodeling, and arrhythmogenesis, and may ultimately lead to aneurysm or thrombus formation [7,8]. In the present case, the initial presentation of a transient ischemic attack underscores how extra-cardiac manifestations may serve as the first indication of underlying cardiomyopathic disease, even when neurological investigations are unremarkable. Importantly, alternative embolic sources were systematically excluded, including significant carotid artery disease, atrial fibrillation on Holter monitoring, and intracranial pathology, thereby reinforcing the plausibility of a cardioembolic origin related to apical stasis.

Transthoracic echocardiography suggested mid-ventricular obstruction through the demonstration of marked hypertrophy and a characteristic systolic “saber-shaped” Doppler profile, a finding consistent with dynamic mid-cavitary gradients reported in MVO-HCM cohorts [8,9]. However, poor apical visualization limited complete assessment of the distal chamber, an inherent limitation in hypertrophic cardiomyopathy when marked wall thickening and interposed lung tissue narrow the acoustic window. Consequently, echocardiography may underestimate mid-cavity obstruction or overlook apical pathology, particularly sequestration, which lacks the dyskinetic, thinned morphology typically associated with apical aneurysm.

Coronary angiography was pursued to clarify the observed apical hypokinesia and surface ECG abnormalities and to exclude epicardial coronary stenosis before attributing the ischemic pattern to MVO-HCM. The absence of obstructive coronary artery disease redirected attention toward non-epicardial mechanisms. Cardiac magnetic resonance (CMR) then proved decisive. CMR delineated severe mid-ventricular hypertrophy with actual apical sequestration, a morphology previously identified as a precursor to apical aneurysm formation and distal chamber ischemia [7-9]. Importantly, the apex in this case remained thick and non-dyskinetic, distinguishing sequestration from a true aneurysm. The presence of intramural mid-ventricular fibrosis and transmural apical late gadolinium enhancement further supported a multifactorial ischemic mechanism arising from elevated distal pressures, microvascular dysfunction, and impaired perfusion reserve rather than epicardial coronary disease [10,11].

The identification of a small mural apical thrombus provided additional diagnostic and prognostic significance. In MVO-HCM, the sequestrated apex is uniquely prone to stasis, and thrombus formation may occur even when global systolic function is preserved. Several reports describe the association between mid-ventricular obstruction, apical aneurysm or sequestration, thrombus, and subsequent embolic complications, including TIA and stroke [7,9,10]. In this context, the patient’s neurologic event likely represented the first clinical manifestation of apical stasis, highlighting the importance of systematic evaluation of the apex in all patients with suspected mid-ventricular obstruction.

From an electrophysiological standpoint, MVO-HCM constitutes a highly arrhythmogenic substrate. Severe hypertrophy, intramural fibrosis, altered intracavitary pressures, and functional apical isolation collectively contribute to an increased propensity for ventricular arrhythmias. In this case, documented non-sustained ventricular tachycardia and extensive fibrosis on CMR substantially elevated the patient’s risk profile. Prior studies have shown that mid-ventricular obstruction with apical involvement carries a particularly high risk of arrhythmias and sudden cardiac death [8,11,12]. Her elevated HCM risk-SCD score, combined with structural and electrical abnormalities, provided strong justification for primary-prevention ICD implantation, fully aligned with contemporary guideline-based risk-stratification strategies [13].

Overall, this case illustrates the diagnostic and prognostic complexity of MVO-HCM with apical sequestration. The patient’s constellation of findings, including TIA, apical thrombus, ischemic injury, NSVT, and distinctive mid-ventricular morphology, reflects the diverse and potentially life-threatening manifestations of this phenotype. This report also emphasizes the need for heightened clinical suspicion, as apical sequestration may remain occult on echocardiography. Comprehensive multimodality imaging and integrated clinical assessment are essential to ensure accurate diagnosis and guide timely and effective management in patients with this challenging and often under-recognized form of hypertrophic cardiomyopathy [7-13].

Several limitations should be acknowledged. The absence of early follow-up CMR at the time of reporting prevents direct assessment of the temporal evolution of the apical thrombus and the stability of the sequestrated apex. Invasive hemodynamic measurements and microvascular perfusion assessment were not performed, limiting precise quantification of distal pressure gradients and the contribution of microvascular dysfunction. Additionally, echocardiographic evaluation of the apex was constrained by suboptimal acoustic windows, illustrating an inherent limitation of transthoracic imaging in this phenotype.

## Conclusions

Mid-ventricular obstructive hypertrophic cardiomyopathy with apical sequestration represents a distinctive and clinically vulnerable phenotype in which structural distortion, impaired distal perfusion, and electrical instability converge. In this patient, multimodality imaging, particularly cardiac magnetic resonance, was essential to uncover the concealed consequences of apical isolation, including ischemic injury and thrombus formation, when initial investigations remained inconclusive. The coexistence of embolic risk, myocardial fibrosis, and documented ventricular arrhythmia further emphasized the need for early, targeted management, including lifelong anticoagulation and prophylactic ICD therapy.

This case highlights the importance of maintaining a high index of suspicion for apical involvement in mid-ventricular HCM, especially in patients with disproportionate ECG abnormalities, unexplained apical hypokinesia, or limited apical visualization on echocardiography. It underscores how timely escalation to advanced imaging can reveal critical pathology that directly influences prognosis, reinforcing the value of comprehensive, individualized assessment in this under-recognized HCM subtype.
